# Does Adulthood Socioeconomic Status Predict Subsequent Telomere Length in Racially and Ethnically Diverse Women?

**DOI:** 10.31586/jbls.2024.1023

**Published:** 2024-09-10

**Authors:** Shervin Assari, Mohammad Dezfuli, Amirreza Peyrovinasab, Hossein Zare

**Affiliations:** 1 Department of Internal Medicine, Charles R. Drew University of Medicine and Science, Los Angeles, CA, United States; 2 Department of Family Medicine, Charles R. Drew University of Medicine and Science, Los Angeles, CA, United States; 3 Department of Urban Public Health, Charles R. Drew University of Medicine and Science, Los Angeles, CA, United States; 4 Marginalization-Related Diminished Returns (MDRs) Center, Los Angeles, CA, United States; 5 Molecular Parasitology Laboratory, Pasteur Institute of Iran, Tehran, Iran.; 6 Tehran Medical Sciences, Islamic Azad University, Tehran, Iran; 7 Department of Health Policy and Management, Johns Hopkins Bloomberg School of Public Health, Baltimore, MD, United States; 8 School of Business, University of Maryland Global Campus (UMGC), Adelphi, MD, United States

**Keywords:** Telomere Length, Poverty, Socioeconomic Status, Biological Aging, Women, Fragile Families and Child Wellbeing Study, Longitudinal Study

## Abstract

**Background:**

Telomere length is a critical biomarker of cellular aging and overall health. While childhood socioeconomic status (SES) indicators such as education and poverty can have long-lasting effects on biological aging, research has shown contradictory results regarding the impact of adulthood SES on future telomere length, particularly in racially and ethnically diverse individuals. This study investigates the effects of baseline adulthood SES indicators such as education and poverty on telomere length nine years later in women, using data from the Future of Families and Child Wellbeing Study (FFCWS).

**Methods:**

We analyzed data from the FFCWS, a longitudinal cohort study. The sample included baseline adulthood SES and follow-up telomere length measure of women (n = 2,421) with varying socioeconomic conditions. Telomere length was measured from saliva samples nine years after the baseline measure of adulthood SES. Education, poverty, and marital status at baseline were assessed. Multivariate linear regression models were used to examine the association between adulthood SES indicators at baseline and future telomere length, controlling for potential confounders.

**Results:**

From the total 2,421 women, 675 were Latino White, 1,158 were non-Latino Black, and 588 were non-Latino White. Our findings indicate that for non-Latino White women poverty at certain level, and childbirth weight, and for non-Latino Black maternal age were predictors of telomere lengths nine years later.

**Conclusion:**

Poverty at a specific level, maternal age and childbirth weight serve as predictors of telomere lengths nine years later in some women. These findings underscore the importance of socioeconomic factors and early-life influences in understanding telomere dynamics and aging processes among women from varied racial and ethnic backgrounds.

## 1. Background

Telomeres are repetitive nucleotide sequences at the ends of chromosomes that protect genetic material from degradation during cell division [1] Over time, telomeres naturally shorten, a process that is accelerated by factors such as oxidative stress, inflammation, and lifestyle behaviors [2] Telomere length is increasingly recognized as a crucial biomarker of cellular aging and overall health [3] Shorter telomeres are associated with numerous adverse health outcomes, including cardiovascular disease, diabetes, and reduced lifespan [4] Understanding the determinants of telomere length, particularly those that can be modified, is essential for addressing health disparities and improving population health.

Childhood and adulthood socioeconomic status (SES) indicators have long been identified as a critical determinant of health, with lower SES linked to a wide range of negative health outcomes [5–7] Poverty, educational attainment, and income exert profounds influences on health through various mechanisms, including access to healthcare, nutrition, exposure to environmental toxins, and stress [8–10] Chronic stress, resulting from financial insecurity, inadequate living conditions, and social disadvantage, can lead to physiological changes that accelerate cellular aging, including telomere shortening [11]

However, research on the association between SES and telomere length is mix. While some research has shown that individuals from lower SES backgrounds tend to have shorter telomeres, highlighting the intersection of socioeconomic factors and biological aging [12] other studies have shown no association between poverty and telomere shortening [13,14]

The relationship between SES indicators and health is multifaceted and operates through a complex interplay of behavioral, psychosocial, and environmental factors [15] People with low SES and those living in poverty are more likely to experience chronic stress, malnutrition, and exposure to harmful environmental conditions, all of which can have lasting effects on their health and development [16–22] These early-life adversities can lead to the dysregulation of physiological systems, including the hypothalamic- pituitary-adrenal (HPA) axis, resulting in elevated cortisol levels and increased oxidative stress [23] These biological changes contribute to telomere shortening and accelerated aging, setting the stage for future health problems.

In addition to early-life experiences, low SES and poverty in adulthood continues to exert significant effects on health [24,25] Adults living in poverty often face barriers to accessing healthcare, leading to delayed diagnoses and inadequate management of chronic conditions [26] They are also more likely to engage in unhealthy behaviors, such as smoking and poor diet, which further exacerbate health disparities [27] The cumulative impact of these factors underscores the importance of considering both early-life and adult SES when examining the determinants of health and aging [28] This study aims to investigate the long-term impact of SES indicators such as educational attainment, poverty, and family structure on telomere length, focusing on a racially and ethnically diverse group of women.

Women, particularly those from lower SES backgrounds, may experience unique stressors and health challenges that can influence their biological aging [29–31] Gender- specific roles and expectations, such as caregiving responsibilities and societal pressures, can lead to chronic stress and mental health issues [32] Women are also more likely to experience poverty and economic instability due to factors such as gender wage gaps, employment in lower-paying jobs, and higher rates of single parenthood [33] These socioeconomic disadvantages can compound the effects of poverty on health, making women an important focus for studies on SES and biological aging [34]

Men and women may be differently susceptible to the adverse effects of low SES and associated chronic stress on telomere length [35] The caregiving role often undertaken by women, combined with economic pressures, can result in prolonged periods of stress [33] leading to greater biological wear and tear. [36,37] Furthermore, women’s health is influenced by a complex interplay of hormonal, genetic, and psychosocial factors that may modify the impact of SES on aging [38] By focusing on women, this study aims to provide insights into how SES indicators such as education, family structure, and poverty interact with gender-specific factors to influence telomere length and overall health.

While there is a growing body of evidence linking SES to telomere length cross- sectionally [11,39–42], significant gaps remain in our understanding of this relationship. Many existing studies are cross-sectional [11,12,41,43–45], limiting our ability to infer causality and understand the long-term effects of SES on biological aging. Additionally, few studies have specifically focused on women [46–48] despite their unique vulnerabilities to the health impacts of SES. Longitudinal research is needed to elucidate how SES influences telomere length over time and to identify potential mediators of this relationship. Finally, few studies have investigated these effects in racially and ethnically minority groups.

This study leverages data from the Future of Families and Child Wellbeing Study (FFCWS) [49–51] a longitudinal cohort study that provides a rich dataset for examining the long-term impacts of low SES on health. The FFCWS includes detailed measures of SES, health behaviors, and biological markers, making it an ideal resource for investigating the links between low SES and telomere length. By focusing on a diverse cohort of women, this study aims to fill critical gaps in the literature and provide a more comprehensive understanding of how socioeconomic factors influence biological aging.

The rationale for this study is grounded in the need to better understand the long- term effects of low SES on biological aging, particularly among women [52] Given the well-documented health disparities associated with lower SES, it is crucial to identify the biological pathways through which low SES influences health [53] Telomere length, as a biomarker of cellular aging, offers a valuable lens through which to examine these effects [2,3,12,13] By investigating the association between SES indicators and telomere length nine years later, this study aims to contribute to the growing body of evidence on SES and health disparities and to inform interventions that can mitigate the adverse effects of poverty on aging.

## 2. Objectives

The primary objective of this study is to investigate the association between SES indicators and telomere length nine years later in women. Specifically, we aim to determine whether women with low SES have shorter telomere lengths compared to those from middle-high SES. By examining these relationships in a longitudinal context, we seek to provide new insights into the long-term impacts of SES on biological aging and to identify targets for interventions aimed at reducing health disparities. We hypothesize that women with low SES will have shorter telomere lengths nine years later compared to those with higher SES. By testing this hypothesis, this study aims to advance our understanding of the biological pathways through which SES influences aging and to inform efforts to promote health equity.

## 3. Methods

### Design and Setting

The Future of Families and Child Wellbeing Study (FFCWS) also known as the Fragile Families and Child Wellbeing Study, is a state-of the art research project that is designed to investigate the challenges faced by economically disadvantaged families in the US. The FFCWS is a birth cohort starting in 1998/2000, following more than 4,000 children from birth to emerging adulthood at age 22 in 2000/2022. More detailed information on the FFCWS study sampling techniques and methods can be found here)[49–51] Here we provide a brief overview of the FFCWS research design.

### Ethics

The study protocol was approved by the Institutional Review Board at Princeton University. Informed consent was obtained from all participating families, with parents or legal guardians consenting on behalf of minors, who also provided their assent. All data collection, storage, and analysis procedures were designed to protect participants' anonymity, and families were compensated for their participation.

### Sample and Sampling

The FFCWS recruited a diverse sample of urban families from 20 major U.S. cities, each with a population exceeding 200,000. The study specifically targeted underrepresented families, particularly non-married, Black, and Latino families. Consequently, the study's sample predominantly consists of low socioeconomic status families, with a substantial representation of Black and Hispanic participants, which does not reflect the overall U.S. population. The analytical sample included 2,421 families with a Black, Latino, or White families. We selected the sample based on availability of telomere length of the mother 9 years after she gave birth.

### Analytical Sample

In the current analysis, we only used the first 9 rather than 22 years of the data.

### Process

Our analysis utilized data from the first and seventh waves of the FFCWS. Socioeconomic position (SEP) data were collected at birth (wave 1, baseline), and the outcome was measured from mother nine years after she gave birth (wave 5). The analysis included 2,421 Black, Latino, and non-Latino White families with follow-up data (telomere length).

### Predictors

Baseline data were collected through interviews with both parents, covering parents' poverty status and family structure at birth. Family poverty status at birth was measured as an ordinal variable ranging from 1 (living under poverty) to 5, with higher number indicator of higher SEP. Poverty categories was calculated based on the household income of the family at baseline. These levels included 1) below 100% of the federal poverty line, 2) below 200% of the federal poverty line, 3) below 300% of the federal poverty line, 4) below 400% of the federal poverty line, and 5) higher than 400% of the federal poverty line. Maternal education was 1) less than high school, 2) high school diploma or equivalent, 3) some college, and 4) college graduate.

### Collection of Saliva Sample

During the Year 9 follow-up wave, saliva was collected from the mother using Oragene DNA Self-Collection Kits (OGR-500) as described for the year 9 follow-up with the following modifications. For those who did not complete a home visit, saliva collection kits were sent to participants via mail and after collection participants returned the kits to Westat via FedEx. Participants were asked not to eat or drink within 30 minutes prior to sample collection. Participants received $20 for completion of the saliva collection [54]

### Telomere Length Measurement

Telomere length (TL; m5_tl) was measured using a quantitative real-time polymerase chain reaction (qPCR) assay. This method uses a double-stranded oligomer standard to measure absolute TL in kilobases (kb) per telomere. An 84-mer double-stranded oligonucleotide with the sequence TTAGGG was used to create a standard curve for telomere quantity, while a 79-mer double-stranded oligonucleotide with a sequence from the 36B4 gene served as the reference gene standard. The efficiency of the PCR plates ranged between 90–110%, and the R2 value for the standard curve exceeded 0.997. TL was calculated by dividing the telomere quantity by the reference gene quantity, then further divided by 92 to determine TL per telomere. Each sample was measured in triplicate for both the telomere and 36B4 primer pairs, and the average of these measurements was used. Samples were randomized across the 96-well plates, which included repeats from previous runs to detect and control for potential batch effects. To address batch effects, reference DNA from two cell lines, one with relatively short telomeres (3C167b) and another with long telomeres (NHFpreT), was included in each run. These cell lines were provided by Dr. Yuanjun Zhao of Pennsylvania State University. The mean TLs for these cell lines in our laboratory are 3.1 kb for 3C167b and 16.8 kb for NHFpreT. Reference DNA was harvested at a single time, aliquoted, and frozen for consistency. The telomere and 36B4 quantities from the reference cell lines were used to normalize variations between runs. For normalization, the geometric mean of the two cell line telomere quantities from each run was divided by the geometric mean of the two cell line telomere quantities from all runs, creating a normalization factor for each run. Each sample's telomere quantity was then divided by its run’s normalization factor, a procedure also applied to the 36B4 quantities. The normalized telomere quantities were divided by the normalized 36B4 quantities to determine telomere length, which was then divided by 92. A replicate DNA sample from volunteers was included in triplicate in all plates, and these measurements were used to compute an inter-run coefficient of variation, which was less than 11% across all runs. We estimated inter-assay intraclass correlation coefficients (ICCs) to assess the consistency and correlation of TL measurements. For statistical analysis, a natural log transformation of the data is recommended to address outliers and correct for the positive skew of the data.

### Statistical Analysis

Data analysis was conducted using Stata version 18.0. Descriptive statistics, including frequencies (percentages) and means (standard deviations), were reported. Bivariate analysis was performed using the Pearson correlation test. For the multivariable analysis, we applied linear regression model. The analysis explored the impact of SES indicators on biological aging of the mothers nine years after they gave birth. We controlled age, race, and ethnicity.

## 4. Results

Overall, 2,421 women with baseline data and telomere length data nine years later entered our analysis. From this number, 675 were Latino White, 1,158 were non-Latino Black, and 588 were non-Latino White. Table 1 shows the association between socioeconomic status at baseline and future telomere length nine years later overall.

Table 2 shows the association between socioeconomic status at baseline and future telomere length nine years later in non-Latino Black participants. For non-Latino Black women in our study, maternal age is a predictor of non-Latino Black’s telomere length in women nine years later.

Table 3 shows the association between socioeconomic status at baseline and future telomere length nine years later in Latino White participants. For Latino White women in the study, no adulthood SES indicators such as poverty status, education, or marital status at baseline were predictive of telomere lengths nine years later.

Table 4 shows the association between socioeconomic status at baseline and future telomere length nine years later in non-Latino White participants. For non-Latino White women in our study, Baby's low birth weight and a poverty threshold of 300% were identified as predictors of future telomere length with statistical significance levels of p < 0.1 and p < 0.05, respectively.

## 5. Discussion

This study investigated the longitudinal association between baseline adulthood SES and telomere length nine years later in women, using data from the FFCWS. The findings showed that some SES factors were predictor of biological aging, for example for non- Latino White women, poverty at a specific level and childbirth weight, and for non-Latino Black women, maternal age, were predictive factors of telomere lengths measured nine years later.

Our findings add to the existing knowledge derived from previous research on the association between SES and telomere length [11,12,14,35,41,43,46,47,55–61]. From these studies, a few have shown that individuals from lower socioeconomic backgrounds exhibit markers of accelerated biological aging, including reduced telomere length. In a series of studies [45,57–60,62,63] Needham has studied associations between SES, stress, telomere length, and mortality. However, very few of these studies have been conducted in a diverse sample of adults, among women, and over time (longitudinal study). Our study extends this literature by using a longitudinal design and focusing specifically on women, however, recruiting a diverse group.

Although we have not measured the causal relationship between low SES and telomere attrition over time. Discrepancies with some cross-sectional studies could be due to differences in sample characteristics or the timing of SES measurements. Our longitudinal approach may help address these issues by capturing the long-term effects of early-life poverty.

Due to multiple mechanisms, we expected an association between low SES and shorter telomere length. Chronic stress is a well-documented pathway through which low SES can impact biological aging [11,44,56,59] Prolonged exposure to stress can lead to dysregulation of the hypothalamic-pituitary-adrenal (HPA) axis, resulting in increased cortisol levels and subsequent telomere shortening [64–66] Additionally, individuals living in poverty often face limited access to healthcare, poor nutrition, and higher exposure to environmental toxins, all of which can contribute to accelerated cellular aging [67,68] Studies have shown that chronic inflammation, oxidative stress, and compromised immune function are more prevalent among those with lower SES, providing plausible biological pathways for the link between poverty and telomere attrition [69,70] Based the findings of our study a wide magnitude of poverty levels in some women may account for the lack of a significant association with a specific poverty threshold.

The focus on women in this study is particularly important, given the unique stressors and societal factors that may exacerbate the effects of poverty on women's health [71] Women often bear a disproportionate burden of caregiving responsibilities, financial stress, and exposure to intimate partner violence, all of which can contribute to chronic stress and biological aging. Furthermore, women may have lower SES than men, due to gender wage gaps and lower overall income levels [72] Our findings highlight the early attention to SES to prevent potential long impact of poverty on low income population.

Intersectionality of race, ethnicity, sex, and SES plays a crucial role in understanding these dynamics, as the combined effects of race, ethnicity, and sex can amplify the impact of poverty on telomere length and aging. Residents of distressed urban areas suffer early aging-related disease and excess mortality. Using a community-based participatory research approach, Geronimus and colleagues collected studied 239 Black, White, and Mexican adults from three Detroit neighborhoods. Telomere length, an indicator of stress- mediated biological aging, was the outcome. Researchers assessed the effects of socioeconomic, psychosocial, neighborhood, and behavioral stressors on telomere length [44]

### Policy Implications

The findings of similar studies on SES determinants of telomere length have significant implications for public health policy and interventions aimed at reducing health disparities. Addressing SES and its associated stressors from an early age are critical for mitigating long-term effects on biological aging. Policies that enhance access to education, healthcare, and social support for low-income families can play a vital role in improving SES and reducing health disparities. Additionally, targeted interventions that focus on women's health, particularly in impoverished communities, are essential for addressing the unique challenges faced by this group. Our study underscores the importance of comprehensive and multifaceted approaches to reduce the burden of poverty and promote health equity. These findings highlight the need for ongoing research to better understand the complex interactions between SES and biological aging and to develop effective strategies for addressing health disparities.

### Strengths and Limitations

This study has several strengths, including its longitudinal design, the use of a well- established cohort, and the focus on a critical biomarker of aging. The longitudinal design enhances the assessment of the relationship between adulthood SES and subsequent telomere length, addressing limitations inherent in previous cross-sectional studies. However, there are also some limitations to consider. Measurement errors in assessing both SES and telomere length could introduce bias into the results. While we adjusted for several confounders, residual confounding from unmeasured variables cannot be completely ruled out. Additionally, the sample sizes varied across racial and ethnic groups of women, which may affect the robustness of subgroup analyses. The study exclusively focused on women (mothers), limiting the generalizability of the findings to similar populations and settings. Future research should aim to replicate these findings in both men and women and explore additional mediators and moderators of the relationship between SES and telomere length.

## 6. Conclusion

The findings suggest that poverty at a specific level, childbirth weight, and maternal age were predictors of telomere lengths nine years later in some women. There is a critical need for policies and interventions that address poverty and its associated stressors to improve long-term health outcomes. By prioritizing efforts to enhance SES and provide targeted support for women in impoverished conditions, we can work towards mitigating the detrimental effects of SES on health and promoting health equity for all.

## Figures and Tables

**Table 1.** Association between socioeconomic status at baseline and future telomere length nine years later overall (women)

	Beta	SE	95%	CI	P
					
Ethnicity (Latino)	0.06	0.02	0.03	0.10	**0.001** ***
Race (Black)	0.04	0.02	0.01	0.07	**0.011** **
Child Sex (Male)	0.00	0.01	−0.02	0.02	0.937
LBW Baby	0.01	0.01	0.00	0.02	0.230
Maternal Age	0.00	0.00	0.00	0.00	**0.096** *
					
Poverty Categories					
Below 100% of the Federal Poverty Line	Ref				
Below 200% of the Federal Poverty Line	0.02	0.02	−0.02	0.06	0.378
Below 300% of the Federal Poverty Line	0.01	0.02	−0.03	0.05	0.564
Below 400% of the Federal Poverty Line	0.01	0.02	−0.03	0.05	0.715
Higher Than 400% of the Federal Poverty Line	0.03	0.02	−0.02	0.07	0.218
					
Maternal Education					
Less than High School	Ref				
Highschool or Equivalent	0.00	0.02	−0.03	0.04	0.756
Some College	0.01	0.02	−0.02	0.05	0.400
College Graduate	−0.01	0.03	−0.06	0.05	0.827
Intercept	1.84	0.03	1.77	1.91	< 0.001***

* p<0.1,

** p<0.05,

*** p<0.001***

**Note:** Data from the Fragile Families and Child Wellbeing Study (FFCWS), Waves 1 and 5 were used. The sample is limited to mothers with telomere length data at Wave 6. LBW stands for Low Birth Weight. All predictors were measured at baseline. The outcome, the natural logarithm of telomere length, was measured at Wave 5 of the study, which represents a 9-year follow-up period.

**Table 2.** Association between socioeconomic status at baseline and future telomere length nine years later in non-Latino Black participants (women)

	Beta	SE	95%	CI	p
					
Child Sex (Male)	−0.01	0.02	−0.04	0.03	0.758
LBW Baby	0.01	0.01	−0.01	0.02	0.514
Maternal Age	0.00	0.00	−0.01	0.00	**0.010** **
					
Poverty Categories					
Below 100% of the Federal Poverty Line	Ref				
Below 200% of the Federal Poverty Line	0.04	0.03	−0.01	0.09	0.150
Below 300% of the Federal Poverty Line	0.03	0.03	−0.02	0.08	0.258
Below 400% of the Federal Poverty Line	0.03	0.03	−0.03	0.08	0.383
Higher Than 400% of the Federal Poverty Line	0.03	0.03	−0.04	0.09	0.435
					
Maternal Education					
Less than Highschool	Ref				
Highschool or Equivalent	0.02	0.02	−0.02	0.06	0.414
Some College	0.04	0.02	−0.01	0.09	0.129
College Graduate	0.01	0.05	−0.09	0.10	0.907
					
Intercept	1.92	0.04	1.84	2.01	< 0.001***

* p<0.1,

** p<0.05,

*** p<0.001

**Note:** Data from the Fragile Families and Child Wellbeing Study (FFCWS), Waves 1 and 5 were used. The sample is limited to mothers with telomere length data at Wave 6. LBW stands for Low Birth Weight. All predictors were measured at baseline. The outcome, the natural logarithm of telomere length, was measured at Wave 5 of the study, which represents a 9-year follow-up period.

**Table 3.** Association between socioeconomic status at baseline and future telomere length nine years later in Latino White participants (women)

	Beta	SE	95%	CI	p
					
Child Sex (Male)	0.02	0.02	−0.02	0.07	0.320
LBW Baby	−0.01	0.02	−0.04	0.03	0.660
Maternal Age	0.00	0.00	0.00	0.01	0.683
					
Poverty Categories					
Below 100% of the Federal Poverty Line	Ref				
Below 200% of the Federal Poverty Line	−0.02	0.04	−0.09	0.05	0.573
Below 300% of the Federal Poverty Line	0.04	0.03	−0.03	0.11	0.246
Below 400% of the Federal Poverty Line	−0.02	0.04	−0.10	0.06	0.623
Higher Than 400% of the Federal Poverty Line	0.02	0.04	−0.06	0.10	0.635
					
Maternal Education					
Less than Highschool	Ref				
Highschool or Equivalent	0.02	0.03	−0.04	0.07	0.571
Some College	−0.03	0.03	−0.09	0.03	0.332
College Graduate	−0.04	0.06	−0.16	0.09	0.579
					
Intercept	1.84	0.06	1.72	1.95	< 0.001***

* p<0.1,

** p<0.05,

*** p<0.001

**Note:** Data from the Fragile Families and Child Wellbeing Study (FFCWS), Waves 1 and 5 were used. The sample is limited to mothers with telomere length data at Wave 6. LBW stands for Low Birth Weight. All predictors were measured at baseline. The outcome, the natural logarithm of telomere length, was measured at Wave 5 of the study, which represents a 9-year follow-up period.

**Table 4.** Association between socioeconomic status at baseline and future telomere length nine years later in non-Latino White participants (women)

	Beta	SE	95%	CI	p
					
Child Sex (Male)	−0.02	0.02	−0.06	0.03	0.522
LBW Baby	0.03	0.01	0.00	0.05	**0.055** *
Maternal Age	0.00	0.00	−0.01	0.00	0.546
					
Poverty Categories					
Below 100% of the Federal Poverty Line	Ref				
Below 200% of the Federal Poverty Line	0.00	0.07	−0.13	0.13	0.964
Below 300% of the Federal Poverty Line	−0.12	0.06	−0.23	−0.01	**0.031** **
Below 400% of the Federal Poverty Line	−0.03	0.06	−0.15	0.08	0.565
Higher Than 400% of the Federal Poverty Line	−0.02	0.06	−0.13	0.09	0.733
					
Maternal Education					
Less than Highschool	Ref				
Highschool or Equivalent	−0.02	0.04	−0.10	0.06	0.638
Some College	0.03	0.04	−0.05	0.12	0.440
College Graduate	−0.01	0.05	−0.11	0.09	0.873
					
Intercept	1.90	0.07	1.75	2.05	< 0.001***

* p<0.1,

** p<0.05,

*** p<0.001

**Note:** Data from the Fragile Families and Child Wellbeing Study (FFCWS), Waves 1 and 5 were used. The sample is limited to mothers with telomere length data at Wave 6. LBW stands for Low Birth Weight. All predictors were measured at baseline. The outcome, the natural logarithm of telomere length, was measured at Wave 5 of the study, which represents a 9-year follow-up period.
